# The Roles of Calmodulin and CaMKII in Cx36 Plasticity

**DOI:** 10.3390/ijms22094473

**Published:** 2021-04-25

**Authors:** Georg R. Zoidl, David C. Spray

**Affiliations:** 1 Department of Biology & Center for Vision Research (CVR), York University, Toronto, ON M3J 1P3, Canada; 2 Dominick P. Purpura Department of Neuroscience & Department of Medicine (Cardiology), Albert Einstein College of Medicine, New York, NY 10461, USA; david.spray@einsteinmed.org

**Keywords:** connexin-36 (*Cx36*), electrical synapse, plasticity, calcium, calmodulin, tubulin, CaMKII

## Abstract

Anatomical and electrophysiological evidence that gap junctions and electrical coupling occur between neurons was initially confined to invertebrates and nonmammals and was thought to be a primitive form of synaptic transmission. More recent studies revealed that electrical communication is common in the mammalian central nervous system (CNS), often coexisting with chemical synaptic transmission. The subsequent progress indicated that electrical synapses formed by the gap junction protein connexin-36 (*Cx36*) and its paralogs in nonmammals constitute vital elements in mammalian and fish synaptic circuitry. They govern the collective activity of ensembles of coupled neurons, and *Cx36* gap junctions endow them with enormous adaptive plasticity, like that seen at chemical synapses. Moreover, they orchestrate the synchronized neuronal network activity and rhythmic oscillations that underlie the fundamental integrative processes, such as memory and learning. Here, we review the available mechanistic evidence and models that argue for the essential roles of calcium, calmodulin, and the Ca^2+^/calmodulin-dependent protein kinase II in integrating calcium signals to modulate the strength of electrical synapses through interactions with the gap junction protein *Cx36*.

## 1. Introduction

The transmission of signals from one neuron to another is primarily via the release of chemicals from the upstream neuron that activates receptors on the downstream neuron. From electron micrographs of close appositions between pre- and postsynaptic elements of the fish brain, JD Robertson proposed the existence of electrical transmission [1], which was demonstrated through electrophysiology recordings (for review, see [2]). Although it was generally believed that so-called electrical or electrotonic synapses were restricted to invertebrates and lower vertebrate nervous systems, it is now clear that they are present in higher mammals, where they are often found in association with chemical synapses (forming “mixed synapses”) and play essential roles in synchronizing inhibitory interneurons, among other functions [3,4]. The molecular identity of the electrical synaptic protein was initially provided from cDNA libraries of skate retina, which predicted a 35-kDa protein that was named, by convention, *Cx35* [5]. Two years later, using the inferior olive as the source material, a new rodent connexin was cloned and subsequently called connexin-36 (*Cx36*) [6]; this protein had 95% sequence identity to skate *Cx35*. Condorelli et al. demonstrated a strong mRNA expression in neurons of the inferior olive, the olfactory bulb, the CA3/CA4 hippocampal subfields, brainstem nuclei of a rat brain, and in the retinal ganglion cell and inner nuclear layers. It was the first connexin found as predominantly expressed in mammalian neurons, and the only other tissue in which it is abundant is the endocrine pancreas [7]. The identification of rat *Cx36* rapidly led to the cloning of other mammalian isoforms of *Cx36* [8], of a group of related connexins in fish [9,10] and in other vertebrates [11]. Following an initial characterization, it became apparent that *Cx36/35* constitutes a novel delta subgroup of connexins. As was predicted by Condorelli et al., molecular cloning of the primary neuronal connexin has enabled the study of its role in the physiology and the pathology of the mammalian brain. In this review, we examine the knowledge gains made in the last few decades. Specifically, we will focus on the emerging role of *Cx36* and the crucial interaction with calcium-activated calmodulin (*CaM*) and calmodulin-dependent protein kinase II (*CaMKII*) partners in the plasticity of electrical synapses.

## 2. Gap Junction Proteins Forming Electrical Synapses between Neurons and Their Interactions with Other Proteins

**Figure 1:**
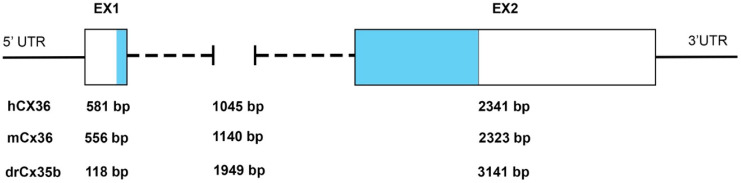
Organization of the exon–intron structure of *Cx35/36* orthologs. Examples of the variable length of Cx35/36 exons (Ex) and introns. The relative position of the protein-coding regions is indicated in light blue. Abbreviations: UTR, untranslated region: *hCX36/GJD2*, *mCx36/Gjd2*, and *drCx35b/gjd2b*. Data source (ENSEMBL releases GRCh38.p13 (human), GRCm39 (mouse), and GRCz11 (zebrafish)).

The initial characterization of mammalian and teleost *Cx36/Cx35* genes showed that the coding sequence is highly conserved. As shown in the amino acid sequence alignment (Supplementary Figure S1), human *Cx36* has 98% identity at the protein level with the mouse and rat *Cx36* and about 70–80% with the ortholog perch, skate, and the zebrafish *Cx35b* proteins [5,9,10]. The gene structure of mammalian Cx36 was somewhat unusual for connexins; the protein-coding region is interrupted by a single intron of variable length, separating the coding region 71 bp after the translation initiation site. Where there is a single *Cx36* protein in mammals, genome duplications in the teleost lineage give rise to multiple paralogs in the zebrafish [12,13]. Each ortholog is presented by two coding exons in all vertebrates, with separating introns of variable lengths (Figure 1).

Mammalian *Cx36* and relatives are expressed in neurons and cells of ectodermic origin, such as pancreatic beta-cells [7], microglia cells [14], and chromaffin cells [15]. Although the *Cx36* regulatory sequences are not well-characterized, the presence of neuron-restrictive silencer elements (NRSE) in the DNA sequence elements of the mammalian and zebrafish promoter region upstream of the coding region has been taken as a reason to explain why *Cx36* is specifically expressed in neurons and in pancreatic beta-cells. NRSEs bind *NRSF/REST*-silencing transcription factors, thereby repressing the transcriptional activity of neural genes in non-neuronal cells. *Cx36* gene expression in insulin-producing beta-cell lines is strictly controlled by the transcriptional repressor *NRSF/REST* [15].

During development, rodent *Cx36* is dynamically expressed over the course of embryonic neurogenesis (for reference, see Supplementary Table S1). *Cx36* localizes to the ventricular zone (VZ) during the first wave of neurogenesis [16]. After birth, the *Cx36* expression is dramatically reduced during postnatal maturation but remains enriched in subsets of principal neurons and interneurons in the inferior olive, hippocampus, olfactory bulb, cerebellum, and thalamus and is sparsely expressed in the cortex. While reports on *Cx36* mRNA expression in rodents’ embryonic brains are detailed, only one study has a reported *Cx35* expression in embryonic and larval zebrafish [17]. The authors described *Cx35* immunoreactivity in the developing brain using a monoclonal antibody raised against perch Cx35. In whole-mount larval zebrafish, from 1 to 15 dpf *Cx35*, immunoreactivity was detected in the developing cerebellum, olfactory bulb, habenula, or the hindbrain.

In the adult mammalian nervous system, the distribution of *Cx36* mRNA examined by in situ hybridization was most intense in the inferior olivary complex, both in principal and accessory nuclei (for reference, see Supplementary Table S2). Moderate labeling was also observed in several myelencephalic nuclei, in specific cells of the cerebellar cortex, in a relatively large subpopulation of cells in the cerebral cortex, in the hilus of the dentate gyrus, and in the strata radiatum and oriens of the hippocampal subfields. Moreover, labeled cells were revealed in all the lamina of the spinal cord gray matter.

After anti-*Cx36* antibodies became available in the early 2000s, rapid advances were made complementing and supplanting the initial RNA-based data. Work from multiple groups demonstrated a widespread localization of the *Cx36* protein in major divisions of the nervous system. For example, *Cx36* was found in the cerebellar cortex, cerebellum, hippocampus, thalamus, the spinal cord, and trigeminal and dorsal root ganglia of the peripheral nervous system [18,19,20,21,22,23,24]. *Cx36* was also discovered in the neurons of the autonomic nervous system [25,26,27]. In sensory systems, *Cx36* is expressed in the retina [28,29,30], olfactory [31,32], and auditory systems [33,34,35,36]. *Cx36* was found in both the interneurons and excitatory neurons [37]. Advances in high-resolution imaging determined the localization of *Cx36* in axo-axonal, axo-dendritic, and dendrodentric contact sites [38]. In confirmed cases, ionotropic and metabotropic NMDA receptors were found in close proximity, suggesting a functional relationship in the post-synapse [23]. *Cx36* was also found in presynaptic contacts at mixed synapses of afferent terminals in the spinal cord [39] or hippocampal mossy fibers [40]. The Nagy group recently identified *Cx36* at the axon initial segments of neurons in the spinal cord, inferior olive, and cerebral cortex [41].

Ultrastructural imaging studies in the 1960s and the 1970s described that gap junctions are found near electron-dense structures. Sotelo et al. (1974) described in their studies on the inferior olive of cats a specialized junctional zone that was named an attachment plate [42]. This structure, sometimes called Nexus or electrical synapse density (ESD), resembles the postsynaptic density found at chemical synapses. Although no comprehensive OMICS study of the molecular components of ESDs has been reported yet, proteins interacting with *Cx36* have been identified. Broadly, these proteins fall into the following classes: cytoskeletal/transport (tubulin [43]); anchoring (*ZO1* family, *AF6*, and *MUPP1* [44,45,46,47,48]); or kinases (*PKA* and *CaMKII* [49,50,51,52,53]); binding proteins (*CaM* [54,55]); and proteins that retrieve *Cx36* from the gap junction plaque [56,57]. Although the rich diversity of the molecules that assemble at electrical synapses parallel the diversity of the proteins involved in forming pre- and postsynaptic compartments at chemical synapses, the molecular and functional relationships of these proteins in mixed synaptic communication have yet to be resolved. In general, the activity of the *Cx36* channels appears closely regulated, notably by posttranslational modifications such as phosphorylation. Protein kinases such as *PKA* and *CaMKII* appear to regulate the gap junction at several levels, including the assembly of channels in the plasma membrane, connexin turnover, and directly affecting the opening and closure (“gating”) of the channels. Further, *Cx36* subunits bind to auxiliary proteins that play essential roles in channel localization and activity or form networks in which these proteins frequently anchor to the cytoskeleton.

The protein complexes at the chemical and electrical synapses share the molecular machinery involving *CaMKII* and *CaM* that achieves functional plasticity [53]. *Cx36, CaM*, and *CaMKII* can all be found in both pre- and postsynaptic compartments, from which it might be assumed that electrical synapses are functionally symmetrical. However, the presence of other proteins can lead to an exciting asymmetry across the synapse. For example, the localization of *NMDA* receptors, a major source of an activity-dependent influx of calcium, in postsynaptic compartments suggests that the *Cx36* signaling complex in the CNS neurons is not a simple mirror image across the gap junction plaque. Additionally, axonal or dendritic transport utilizes distinct mechanisms. Together, there is growing evidence for functional asymmetry potentially arising from differences in posttranslational modifications of individual *Cx36* proteins [49] by asymmetrically organized signaling complexes of *Cx36*-associated proteins found at the ESD [44,47,58], as well as by asymmetric properties of pre- and postsynaptic membranes (for review, see [59,60]).

## 3. Cx36 Interaction with Calmodulin Connects Calcium Signals with Gap Junction Communication

**Figure 2:**
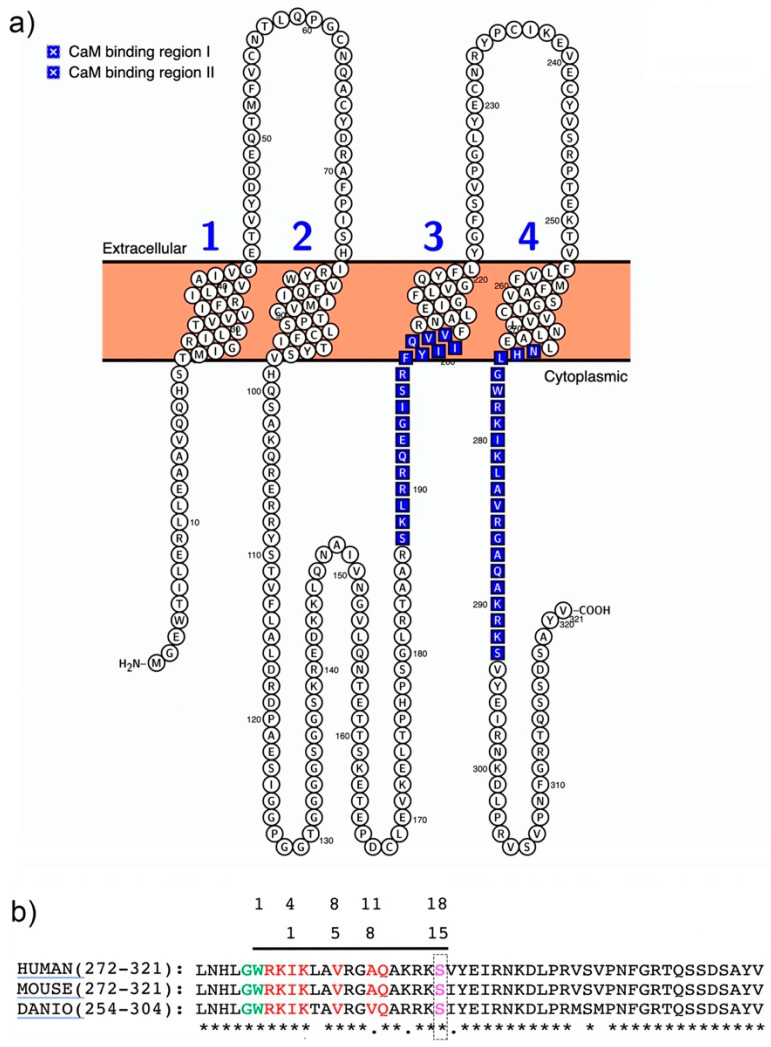
Structure of the *Cx36–CaM*-binding motifs. (**a**) Visualization of the predicted murine *Cx36* structure using Protter software [73]. The *CaM*-binding regions I and II are indicated in blue. (**b**) Protein sequence alignment of the carboxyterminal domains of mammalian and zebrafish (*Cx35b*). The sequence homology and the two overlapping *CaM*-binding motifs described by [54,64] are highlighted in light green and red. The *PKA* phosphorylation site in position S293 (pink) is located at the boundary of the core *CaM-*binding motif [50].

The importance of calmodulin-regulated gap junction communication has been reviewed recently [61,62,63]. In the nervous system, calcium (Ca^2+^) is an essential ligand that binds its primary intracellular receptor calmodulin (*CaM*) to trigger multiple downstream processes and pathways in both pre- and postsynaptic compartments. *CaM* binding to *GST–Cx36* fusion proteins was first demonstrated for mammalian *Cx36* and fish orthologs and involved the C-terminal domains [54,55] (Figure 2a). Recently, a second *CaM*-binding site was found in the cytoplasmic loop of the perch *Cx35*. This site was not analyzed in detail after showing weak binding characteristics and rapid kinetics in surface plasmon resonance experiments [64]. The in vitro binding and dissociation kinetics were in the µM concentration range and Ca^2+^-dependent. The rapid on and off binding rates implies that the interaction of *Cx36* with *CaM* may change dynamically in neurons when transient increases of Ca^2+^ occur in or near the activated synapses. Siu et al. [54] demonstrated in Neuro2a cells that Ca^2+^-loaded *CaM* binds a site in the carboxy-terminus of *Cx36* overlapping with a previously identified CaMKII-binding site [52,53]. This extended the findings by Burr et al. [55], who argued that the *CaM*-binding site’s characteristics might confer sensitivity to the competition with other proteins with higher CaM affinities.

It can be reasoned that local differences in the intracellular calcium concentrations open up ample possibilities for the spatial and temporal sequestering of interactions between *Cx36* and *CaM*. This was demonstrated in Neuro2a cells, where *Cx36* was able to bind *CaM* outside of the gap junction plaque in the ER/Golgi complex [54]. Similarly, another connexin, *Cx32*, interacts with *CaM* before GJs are formed [65]. The binding of Cx proteins to *CaM* has diverse functional outcomes. In Neuro2a cells, Siu et al. (2016) showed that Ca^2+^-loaded *CaM* increased the coupling across GJs formed *by Cx36* channels. This outcome is different from other connexins closed after binding [66,67]. The NMR solution structure demonstrated that *CaM* binds *Cx36* in a compact state with significant hydrophobic contributions from amino acid W277 at anchor position 1 and V284 at position 8 of *Cx36* [54] (Figure 2b). Different functional outcomes of *Cx36* binding to *CaM* were reported by Aseervatham et al. [64] in Hela cells expressing *Cx35*, where *CaM* binding induced Ca^2+^-mediated uncoupling. The opposite effect on the coupling is surprising, since both studies used site-directed mutagenesis and the pharmacological blocking of *CaM*. In general, Hela cells and Neuro2a cells are not thought to express significant amounts of endogenous connexins or form gap junctions. However, coupling in HeLa cells is documented [68,69], which could confound the interpretation of the results. It stands to be demonstrated whether the different intracellular environments and degrees of endogenous gap junction coupling of human cervical carcinoma (Hela) and rodent neuroblastoma (Neuro2a) cell lines account for the reported opposite outcomes.

The study by Aseervatham et al. [64] further indicated that the *CaM*-binding motif in the carboxyterminal domain of *Cx35/Cx36* allows for structural flexibility. Aseervatham et al. reported a 1-5-10-binding motif, which is distinct from the previously reported 1-4-8-11 motif [54]. The differences between the two predicted *CaM*-binding motifs may reflect the distinct computational and analytic approaches used in separate studies. Equally well, the *CaM*-binding motifs of *Cx35/6* could also undergo the rapid structural adaptation of protein interfaces that adjust the binding properties when local calcium concentrations are dynamic. In favor of this argument are the rapid structural adaptations of CaM, which were exploited to develop fast-acting and sensitive biosensors for intracellular calcium dynamics. Furthermore, the *Cx36* carboxyterminal domain structure is predicted to be intrinsically disordered, like other connexins [67,70,71].

The use of alternative *CaM*-binding motifs might also prepare *Cx36* for different protein interactions. An example is the interaction of *Cx36* with tubulin. The *Cx3*6-binding motif overlaps with the *CaM*-binding site but is distinct from those found in *Cx43* [43,72]. The mechanistic details of the trafficking to the gap-junction plaque (GJP) were demonstrated using a pharmacological and mutational analysis, demonstrating that amino acids K279, I280, and K281 were critical. The potentiation of the communication strength between coupled cells was demonstrated, reinforcing the role of and protein transport to fine-tuned gap junction communication.

## 4. Building a Cx36-CaMKII Interaction Complex

**Figure 3:**
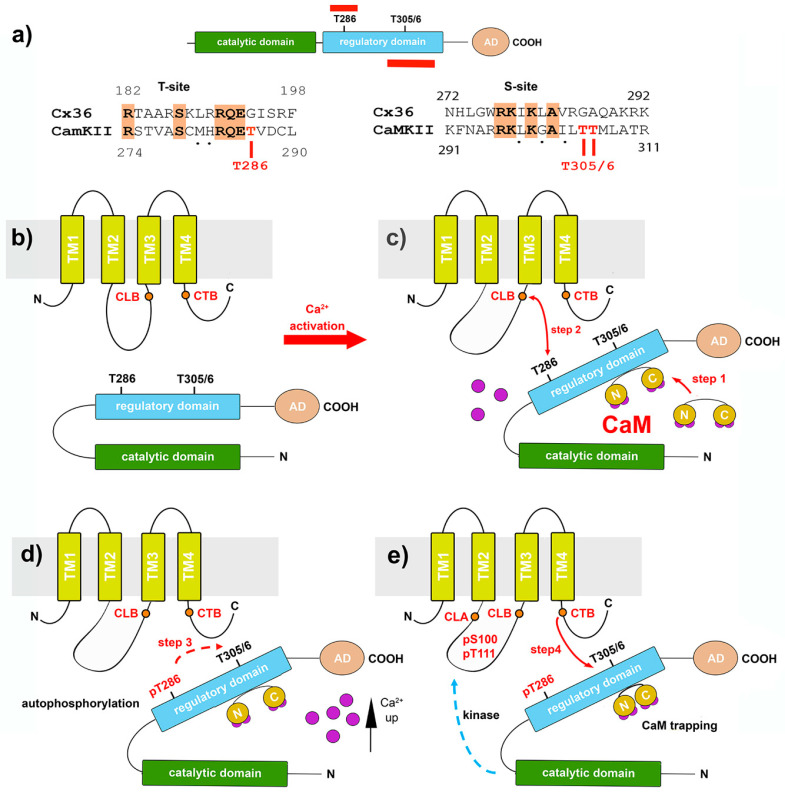
The *Cx36–CaMKII*-binding complex. (**a**) The catalytic, regulatory, and accessory domain of *CaMKII*. The autophosphorylation and T-site (top) and the *CaM*-binding site at the S-site are shown (bottom) in red. T286 and T305/6 indicated phosphorylation sites in the regulatory domain. The sequence alignments below show conserved amino acids in the T- and S-sites of *Cx36* and *CaMKII*. (**b**–**e**) Model of the activation sequence in which *CaMKII* is opened by calcium-activated *CaM*, followed by the sequential binding and phosphorylation of *Cx36* (modified from reference [53]). (**c**) In step 1, elevated intracellular Ca^2+^ activates *CaM*, and the binding of *Ca^2+^/CaM* to the autoinhibitory domains displaces the gate and, in step 2, allows the *CaMKII*-binding domain in the cytoplasmic loop of *Cx36* (CLB) to interact with the kinase’s target site, *CaMKII*. (**d**) Autophosphorylation at T286 (step 3) (**e**) enables the kinase to bind to a domain in the carboxyl terminus of *Cx36* (labeled CTB) (step 4). Phosphorylation of the cytoplasmic loop sites follow (dashed blue arrow in (**e**)), as well as in the carboxyl terminal domain.

The research summarized in the previous section established *Cx*36 as a hub binding Ca^2+^-loaded *CaM*, and they identified this interaction as a critical step with implications for functions preceding the initiation of calcium/calmodulin-dependent kinase II (*CaMKII*)-mediated plasticity at electrical synapses. Here, we will demonstrate that the molecular interaction of *Cx36* with *CaMKII* at electrical synapses has a resemblance to interactions of *CaMKII* with *NMDA* receptors, enabling chemical synaptic potentiation, which is at the core of plasticity and learning and memory [74,75]. *CaMKI*I is a dodecameric holoenzyme [76]. *CaMKII* comprises a family of >25 isoforms that are derived from four genes (α, β, γ, and δ). The α- and β-subunits are the predominant isoforms in the brain, where they form holoenzymes that are composed of either one or both subunit types [77]. Each *CaMKII* isoform consists of a catalytic domain, an autoinhibitory domain, a variable segment, and a self-association domain (Figure 3a). The catalytic domain is responsible for the kinase activity of *CaMKII* [78,79]. The regulatory domain is required for autophosphorylation and *CaM* binding [80]. The association domain forms a hub-like, tetrameric assembly, composed of two rings of seven protomers each, which are stacked head-to-head and held together by extensive interfaces [81,82].

Central to the ability of *CaMKII* to generate sustained changes in the postsynaptic efficacy after stimulation is the interaction of the subunits with the *Ca^2+^/CaM* complex. In the “off state”, *CaMKII* is inactive, because the enzyme’s autoinhibitory regulatory domain buries the catalytic region, preventing autoactivation and substrate binding (Figure 3b) [83,84]. Interestingly, both the autoactivation (T-site) and substrate binding (S-site) domains of *CaMKII* share sequence homology with sequences in the cytoplasmic loop (CLB) and carboxyterminal (CTB) domain of *Cx36* [53]. The binding of *Ca^2+^/CaM* releases CaMKII from autoinhibition by exposing the T-site and the S-site of the enzyme. This opening of the *CaMKII* generates autonomous kinase activity by an intraholoenzyme, causing intersubunit phosphorylation of the threonine residue at position T286 [79,80,85]. Once phosphorylated at T286, the regulatory domain with its T- and S-sites can no longer interact with the catalytic site’s corresponding domains, allowing the catalytic domain to access and phosphorylate substrates [86]. At this step, sustained autonomous *CaMKII* activity is independent of *Ca^2+^/CaM* and keeps the kinase active after the initial Ca^2+^ stimulus has subsided.

One of the most prominent substrates of *CaMKI*I at postsynaptic sites is the *NR2B* subunit of the *NMDA* receptor. The autophosphorylation-dependent binding of *CaMKII* to *NR2B* locks *CaMKII* in the active conformation [87,88]. *CaMKII* binding to the *NR2B* protein requires two binding sites [75,89]. Based on similarities of the interaction of the *Cx36* and *CaMKII*-binding sites with properties of the *NR2B* subunit, a model was proposed where the CLB and CTB sites of *Cx36* function in a bipartite fashion, like the interaction of the *NR2B* subunit with *CaMKII* [53]. The proposed sequence of events put forward in Figure 3b–e is based on the autoinhibitory “gate” hypothesis of the regulatory and catalytic domains of *CaMKII*. The following sequence of events was predicted. In step 1, the binding of *Ca^2+^/CaM* to the autoinhibitory domains displaces the gate and allows the CLB domain (step 2) to interact with the kinase’s target site. The phosphorylation of *CaMKII* is not required for this initial step. The subsequent autophosphorylation at T286 (step 3) enables the kinase to interact with the target or substrate proteins and bind Cx36–CTB to its corresponding substrate recognition motif (step 4). An argument for the subsequential processing of both *Cx36* segments during interactions with the kinase can be made from the discovery of phosphorylation found at amino acid residues S110 and T111 within the cytoplasmic loop (CLA) and at S315 of the carboxyl-terminal domain [53]. Based on the model shown, it is reasonable to speculate that the phosphorylation of S110/T111 is a direct consequence of fully activating *CaMKII*, even though the exact timing has not been determined (steps 3 and 4). Although *CaMKII* does not phosphorylate *Cx36* at position S315 in vitro, this modification is notable. At S315, *Cx36* is phosphorylated by *PKA* in a position where structural changes after phosphorylation could affect the dynamic coupling–uncoupling of *Cx36* from the interaction complexes. This effect would explain the antagonistic nature of *CaMKII* and *PKA* phosphorylation on *Cx36*-mediated gap junction coupling in the cerebellum [49] or the rodent [50] or zebrafish retina [51,90].

Having established that phosphorylation by *PKA* and *CaMKII* keeps *Cx36* coupling and uncoupling in a physiological range, the next question arises as to what the role is of the two *CaMKII*-binding regions. It has been demonstrated that the binding and extent of phosphorylation of the *Cx36* cytoplasmic domains varied according to the autophosphorylated state of *CaMKI*I and, thus, resembled the behavior of fragments of the *NR2B* subunits of the NMDA receptor. The autophosphorylation of *CaMKII* is less effective at the Cx36–CLB site in terms of binding and phosphorylation. The Cx36–CTB site exhibited a higher efficacy toward both effects when *CaMKII* was autophosphorylated. Since *CaM* bound to the CT of *Cx36* would inhibit the interaction with the substrate-binding site of the kinase, the several orders of magnitude increase in affinity of *CaMKII* to *Ca^2+^/CaM* after autophosphorylation of T286 (so-called *Ca^2+^/CaM* trapping) (Figure 3e), could explain how *CaM* competes with *Cx36* for the overlapping CTB-binding motif. It suggests that when *CaM* is displaced from the CTB site, *Cx36* is released from its inhibitions and able to bind to *CaMKII*.

**Figure 4:**
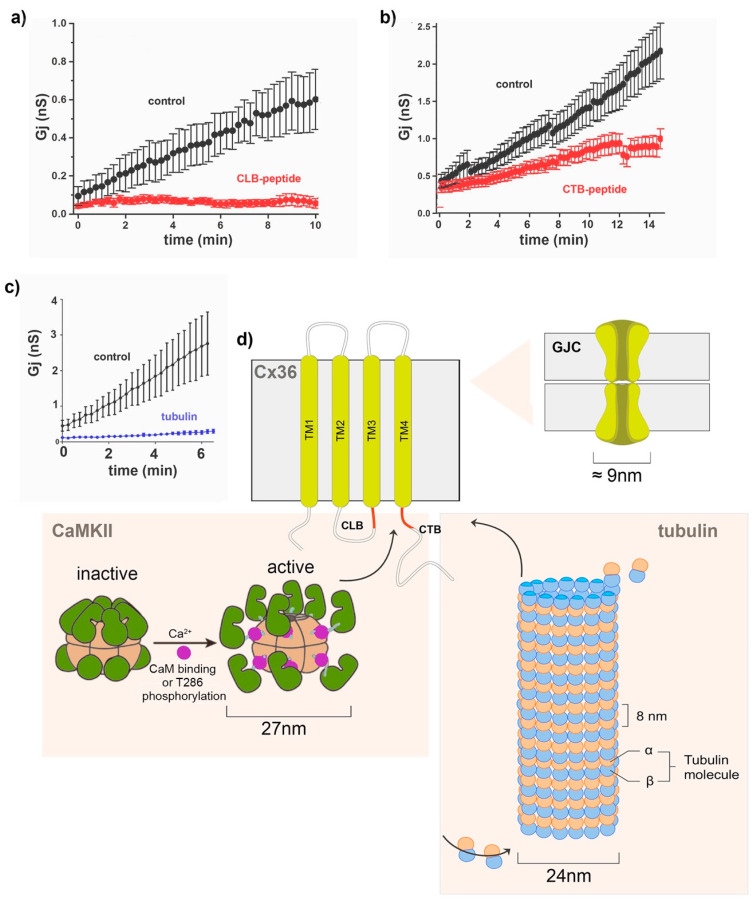
*Cx36* interactions with macromolecular protein complexes. (**a**–**c**) Dual patch–clamp recordings of Neuro2a cell pairs expressing *Cx36* typically reveal an initial 5–10-fold increase in the junctional conductance (Gj), termed run-up (black symbols), modified from reference [52]. The extent of the run-up was reduced by the intracellular application of peptides (100 µM) blocking the CLB (aa175–195) and CTB (aa272–292) sites of *Cx36* (curves with red symbols). (**c**) The intracellular application of monomeric tubulin (50 µM) reduced the run-up plasticity (blue trace). Modified from reference [43]. (**d**) Summary of macromolecular interactions between *Cx36* and the *CaMKII* holoenzyme or microtubules. As illustrated in Figure 3, *CaMKII* is activated by binding to the *Ca^2+^/CaM* complex or following T286 phosphorylation (modified from reference [85]. Active *CaMKII* binds to the intracellular CTB domain, a binding target shared with the *CaM* and microtubules. Details of the spatiotemporal regulation of how the macromolecular complexes bind are presently unknown; however, the linkage of *Cx36* to microtubules likely facilitates its delivery to [87] the GJP, a function that is lost when microtubules depolymerize.

The initial electrophysiological studies of *Cx36* revealed that the gap junction channels have a remarkably low conductance and are highly insensitive to transjunctional voltage compared to other connexins [91,92]. These properties optimize transmissions during significant voltage changes and endow the electrical synapses with the requirement that substantial changes in the conductance are accompanied by a significant increase or decrease of the number of open or closed channels. A substantial increase in junctional conductance was recorded between pairs of voltage-clamped cells after the whole-cell mode was achieved, generally rising about tenfold for ten minutes [52,93] (see Figure 4a–c, black traces). This phenomenon of adaptive plasticity was coined a “run-up”. It is unique for cell pairs expressing *Cx36* tested in Neuro2a cell cultures [52]. It illustrated the time-dependent changes in *Cx36* junctional conductance (Gj) and was explained as the increased open probability following altered phosphorylation or transport of *Cx36* to the gap junction plaque (GJP). Experimental ablation of the CLB and CTB-binding regions in *Cx36* (Figure 4a,b) [52] or inhibition of *CaMKII* was used to test for a weakening of the “run-up” phenomenon previously described [93]. In these studies, the run-up was virtually absent in mutants lacking the *CaMKII*-binding regions. It was proposed that the run-up process involved *CaMKII* increasing the channel open time rather than affecting the channel insertion internalization [52]. The pharmacological blockade of *CaMKII* using KN-93 and with cognate peptides applied intracellularly, together with site-directed mutagenesis of the previously described *CaMKII*-binding and phosphorylation sites of *Cx36*, connected the previously reported direct interaction of *CaMKII* with its binding sites CLB and CTB, as well as the *CaMKII* phosphorylation sites at S110/T111 of Cx36 [53].

The control of Cx36 Gj was not restricted to the interaction with *CaMKII*. Brown et al. [43] determined that *Cx36* binds to tubulin using a motif overlapping with the afore-described *CaM* and *CaMKII*-binding motifs. Dual patch–clamp recordings demonstrated that pharmacological interference of the cytoskeleton abolished *Cx36* plasticity (Figure 4c). Mechanistic details of *Cx36* trafficking demonstrated that tubulin-dependent transport potentiates the synaptic strength by delivering channels to GJPs. The extent to which the microtubule-mediated delivery in *Cx36* accounts for the run-up compared to other stimuli remains to be determined.

The binding of three calcium-dependent proteins to overlapping domains of *Cx36* emphasizes that the plasticity of the electrical synapses is controlled at multiple levels and that local calcium fluctuations are a critical factor in the processes. It also points out that these interactions involve binding hexametric gap junction channels to the macromolecular complexes formed by the *CaMKII* holoenzyme or the microtubule superstructure (Figure 4d). While structural and mechanistic constraints imposed by interactions of these large protein assemblies remain to be fully resolved, the machinery, protein–protein interactions and post-translational modifications involved in long-term electrical synapse changes may be as complex as those at chemical synapses.

As pointed out by reference [64], gap junctions formed by *Cx36* that function as electrical synapses within networks of neurons will routinely encounter large fluctuations in the local cytoplasmic calcium concentration. Presently, we have only started to uncover the multi-faceted details of how dynamic calcium signaling modulates the functions of mixed synapses. The complexity of the problem of studying calcium signals separated in time and space is immense. Postsynaptic dendritic spines are home to >1000 different proteins, many of which are calcium-dependent and require demand transport and activation, such as *AMPA/NMDA* receptors or *Cx36*. The molecular composition of presynaptic contacts is equally challenging. Sourcing calcium and building often short-term (ms) to second signals requires mechanisms not fully detailed yet. The elevation of intracellular calcium can arise from multiple sources, such as voltage-gated calcium channels, *AMPA/NMDARs*, or the axonal or spine ER. Identifying the relevant spatiotemporal calcium signals that drive *Cx36*-mediated plasticity will require a new generation of tools that combine the sensitivity of genetically encoded biosensors with advanced and dynamic high-resolution microscopy. The O’Brien group’s recent work is a significant step in the direction needed [94]. In this work, the investigators demonstrated how changes in the local Ca^2+^ signaling regulates plasticity using a GCaMP Ca^2+^ biosensor fused to *Cx36*. The *Cx36–GCaMP* biosensor robustly reported local Ca^2+^ increases in response to the addition of a Ca^2+^ ionophore with increases in the fluorescence that recovered during washout. Recovery was strongly dependent on Na^+^-Ca^2+^ exchange activity. *Cx36–GCaMP* revealed transient and concentration-dependent increases in local Ca^2+^ after the application of glutamate in cells transfected with *NMDA* receptor subunits. The mechanism was dependent, in part, on the *CaMKII* activity. In summary, *Cx36–GCaMP* is an effective tool to measure the changes in the Ca^2+^ microenvironment around the *Cx36* gap junctions. Based on this framework, it seems reasonable to build a toolbox of customized genetically encoded fluorescent biosensors or FRET sensors to dissect the calcium signaling pathways in the context of *Cx36* microdomains to shed light on the mechanistic details of how electrical synapses operate. Finally, the recent advances in cryoEM suggest that resolving the structure of *Cx36–CaM* or *Cx36–CaMKII* in its different stages, in similar detail as the recently reported structures of pannexins and innexins, is a timely objective.

## 5. Conclusions

Regardless of the lack of a precise understanding of the conformational changes underlying the events described above, *Cx36* might quickly sample *CaM*, *CaMKII*, and other protein-mediating functions such as transport or maintaining the local scaffold. Primed by local calcium dynamics, *CaM* may translate the numerous signaling contexts *Cx36* encounters throughout the passage from the ER/Golgi complex to the synaptic contacts. Beyond doubt, the decoding of calcium signals is essential as proteins stimulated by the calmodulin–calcium complex are protein kinases, phosphatases, or accessory proteins with multiple functions. How these complex interactions are governed is presently unknown.

## Data Availability

Not applicable.

## Supplementary Materials

The following materials are available online and can be found at https://www.mdpi.com/article/10.3390/ijms22094473/s1: Supplementary Figure S1; Supplementary Table S2: Cx36 mRNA expression during early development of the mouse brain; Supplementary Table S2: Cx36 mRNA and protein expression in the adult rodent nervous system.
