# Supplementary material for: The Roles of Calmodulin and CaMKII in Cx36 Plasticity
Source: Int J Mol Sci. 2021 Apr 25;22(9):4473. doi: 10.3390/ijms22094473 (PMC8123330; doi:10.3390/ijms22094473)
Supplement: Supplementary file 1 [file ijms-22-04473-s001.zip › ijms-1181395-supplementary.pdf]

Supplementary Figure S1: Top) Cladogram of *Cx35/36* protein sequences representing mammalian and lower vertebrates used as animal models in neurobiology research. **Bottom)** Multiple sequence alignment using Clustal Omega with default settings at the European Bioinformatics Institute (EBI). Homology regions for *CaM/CaMKII*-binding and S110/T111 phosphorylation in red.

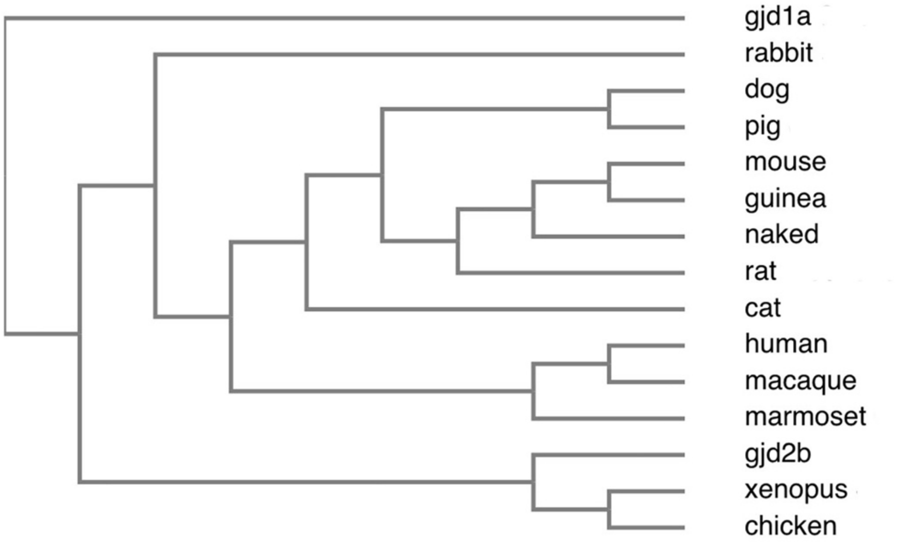


```
gjd1a          MGEWTILERLLEAAVQQHSTMIGRILLTVVVIFRILIVGIVGEKVYEDEQIMFICNTLQP   60
rabbit         MGEWTILERLLEAAVQQHSTMIGRILLTVVVIFRILIVAIVGETVYDDEQTMFVCNTLQP   60
dog            -------ERLLEAAVQQHSTMIGRILLTVVVIFRILIVAIVGETVYDDEQTMFVCNTLQP   53
mouse          MGEWTILERLLEAAVQQHSTMIGRILLTVVVIFRILIVAIVGETVYDDEQTMFVCNTLQP   60
rat            MGEWTILERLLEAAVQQHSTMIGRILLTVVVIFRILIVAIVGETVYDDEQTMFVCNTLQP   60
human          MGEWTILERLLEAAVQQHSTMIGRILLTVVVIFRILIVAIVGETVYDDEQTMFVCNTLQP   60
macaque        MGEWTILERLLEAAVQQHSTMIGRILLTVVVIFRILIVAIVGETVYDDEQTMFVCNTLQP   60
marmoset       MGEWTILERLLEAAVQQHSTMIGRILLTVVVIFRILIVAIVGETVYDDEQTMFVCNTLQP   60
cat            MGEWTILERLLEAAVQQHSTMIGRILLTVVVIFRILIVAIVGETVYDDEQTMFVCNTLQP   60
pig            MGEWTILERLLEAAVQQHSTMIGRILLTVVVIFRILIVAIVGETVYDDEQTMFVCNTLQP   60
guinea         MGEWTILERLLEAAVQQHSTMIGRILLTVVVIFRILIVAIVGETVYDDEQTMFVCNTLQP   60
naked          MGEWTILERLLEAAVQQHSTMIGRILLTVVVIFRILIVAIVGETVYDDEQTMFVCNTLQP   60
gjd2b          MGEWTILERLLEAAVQQHSTMIGRILLTVVVIFRILIVAIVGETVYDDEQSMFVCNTLQP   60
xenopus        MGEWTILERLLEAAVQQHSTMIGRILLTVVVIFRILIVAIVGETVYDDEQTMFVCNTLQP   60
chicken        MGEWTILERLLEAAVQQHSTMIGRILLTVVVIFRILIVAIVGETVYDDEQTMFVCNTLQP   60
                      *******************************.****.**:*** **:******

                                                                S110/T111
                                                                   --
gjd1a          GCNQACYDKAFPISHIRYWVFQIILVCTPSLCFITYSVHQSAKHKDQRYTLLHGPY----   116
rabbit         GCNQACYDRAFPISHIRYWVFQIIMVCTPSLCFITYSVHQSAKQRERRYSTVFLSLDRDP   120
dog            GCNQACYDRAFPISHIRYWVFQIIMVCTPSLCFITYSVHQSAKQRERRYSTVFLALDREP   113
mouse          GCNQACYDRAFPISHIRYWVFQIIMVCTPSLCFITYSVHQSAKQRERRYSTVFLALDRDP   120
rat            GCNQACYDRAFPISHIRYWVFQIIMVCTPSLCFITYSVHQSAKQRERRYSTVFLALDRDP   120
human          GCNQACYDRAFPISHIRYWVFQIIMVCTPSLCFITYSVHQSAKQRERRYSTVFLALDRDP   120
macaque        GCNQACYDRAFPISHIRYWVFQIIMVCTPSLCFITYSVHQSAKQRERRYSTVFLALDRDP   120
marmoset       GCNQACYDRAFPISHIRYWVFQIIMVCTPSLCFITYSVHQSAKQRERRYSTVFLALDRDP   120
cat            GCNQACYDRAFPISHIRYWVFQIIMVCTPSLCFITYSVHQSAKQRERRYSTVFLTLDREP   120
```

```
pig             GCNQACYDRAFPISHIRYWVFQIIMVCTPSLCFITYSVHQSAKQRERRYSTVFLALDRDP   120
guinea          GCNQACYDRAFPISHIRYWVFQIIMVCTPSLCFITYSVHQSAKQRERRYSTVFLALDRDA   120
naked           GCNQACYDRAFPISHIRYWVFQIIMVCTPSLCFITYSVHQSAKQRERRYSTVFLALDRDA   120
gjd2b           GCNQACYDKAFPISHIRYWVFQIIMVCTPSLCFITYSVHQSAKQKERRYSTIYLSLDKDP   120
xenopus         GCNQACYDRAFPISHIRYWVFQIIMVCTPSLCFITYSVHQSAKQRERRYSTVFLTVDRDP   120
chicken         GCNQACYDQAFPISHIRYWVFQIIMVCTPSLCFITYSVHQSAKQRERRYSTVFLTLERDQ   120
                ********:***************:******************::::**: :.

gjd1a           --------IDHGHG------PSRKL--RNINGILVHPE---SKDDRECLDLKDIPNIPAG   157
rabbit          PDSMGGPGGTGGGGSGGGKREDKKLQNAIVNGVLQNPENPSKETEPDCLEVKELTPHPSG   180
dog             PESMGGPGGTGSGASGSSKREDKKLQNAIVNGVLQNTENTSKEVEPDCLEVKELTPHPSG   173
mouse           AESIGGPGGTGGGGSGGSKREDKKLQNAIVNGVLQNTETTSKETEPDCLEVKELTPHPSG   180
rat             AESIGGPGGTGGGGSGGSKREDKKLQNAIVNGVLQNTETTSKETEPDCLEVKELAPHPSG   180
human           PESIGGPGGTGGGGSGGGKREDKKLQNAIVNGVLQNTENTSKETEPDCLEVKELTPHPSG   180
macaque         PESIGGPGGTGGGGSGGGKREDKKLQNAIVNGVLQNTENTSKETEPDCLEVKELTPHPSG   180
marmoset        PESIGGPGATGGGGSGGGKREDKKLQNAIVNGVLQNTENTSKETEPDCLEVKELTPHPSG   180
cat             PESMGGPGGTGGGGSGGGKRDDKKLQNAIVNGVLQNTENTSKETEPDCLEVKELTPHPSG   180
pig             PESMGGPGGTGGGGSGGGKREDKKLQNAIVNGVLQNTENTSKETEPDCLEVKELTPHPSG   180
guinea          PESMGGPGGTGGGGSGGGKREDKKLQNAIVNGVLQNTENTSKETEPDCLEVKELTPHPSG   180
naked           PESMGGPGGTGGGSSGGGKREDKKLQNAIVNGVLQNTENTSKETEPDCLEVKELTPHPSG   180
gjd2b           DTMR--------------RDDSKKIKNTIVNGVLQNTENSTKESEPDCLEVKEIPN---S   163
xenopus         DTVK--------------REDSKKIKNTLVNGVLQNTENSTKEAEPDCLEIKEIPN---P   163
chicken         DSMK--------------REDSKKIKNTIVNGVLQNTENSTKEAEPDCLEVKEIPN---P   163
                                     .:*:    :**:* : *   .: : :**::*::
```

T-site homology region

```
                  ---------------

gjd1a           VTYSKSAKIRQQEGISRFYVIQVVFRNVLEIGFLAGQYFLYGFNVPAMFECDRYPCVKEV   217
rabbit          MRTAAKSKLRRQEGISRFYIIQVVFRNALEIGFLVGQYFLYGFSVPGLYECNRYPCIKEV   240
dog             LRTAARSKLRRQEGISRFYIIQVVFRNALEIGFLVGQYFLYGFSVPGLYECDRYPCIKEV   233
mouse           LRTAARSKLRRQEGISRFYIIQVVFRNALEIGFLVGQYFLYGFSVPGLYECNRYPCIKEV   240
rat             LRTAARSKLRRQEGISRFYIIQVVFRNALEIGFLVGQYFLYGFSVPGLYECNRYPCIKEV   240
human           LRTASKSKLRRQEGISRFYIIQVVFRNALEIGFLVGQYFLYGFSVPGLYECNRYPCIKEV   240
macaque         LRTASKSKLRRQEGISRFYIIQVVFRNALEIGFLVGQYFLYGFSVPGLYECNRYPCIKEV   240
marmoset        LRTASKSKLRRQEGISRFYIIQVVFRNALEIGFLVGQYFLYGFSVPGLYECNRYPCIKEV   240
cat             LRTAARSKLRRQEGISRFYIIQVVFRNALEIGFLVGQYFLYGFSVPGLYECDRYPCIKEV   240
pig             LRTAARSKLRRQEGISRFYIIQVVFRNALEIGFLVGQYFLYGFSVPGLYECDRYPCIKEV   240
guinea          LRTAARSKLRRQEGISRFYIIQVVFRNALEIGFLVGQYFLYGFSVPGLYECNRYPCIKEV   240
naked           LRTAARSKLRRQEGISRFYIIQVVFRNALEIGFLVGQYFLYGFSVPGLYECNRYPCIKEV   240
gjd2b           AMRTTKSKMRRQEGISRFYIIQVVFRNALEIGFLVGQYFLYGFNVPAVYECDRYPCIKDV   223
xenopus         SIRTTKSKMRRQEGISRFYIIQVVFRNALEIGFLVGQYFLYGFNVPSMYECDRYPCIKEV   223
chicken         AIRTTKSKMRRQEGISRFYIIQVVFRNALEIGFLVGQYFLYGFNVPSMYECDRYPCIKEV   223
                   :  :*:*:********:*******.******.********.**.::**:****:*:*
```

S-site homology region

```
                                                  ----------------

gjd1a           ECYVSRPTEKTVFLVFMFAVSGICVVLNLAELNHLGWRKIKTAIRGVQARRKSICEIRKK   277
rabbit          ECYVSRPTEKTVFLVFMFAVSGICVVLNLAELNHLGWRKIKLAVRGAQAKRKSVYEIRNK   300
dog             ECYVSRPTEKTVFLVFMFAVSGICVVLNLAELNHLGWRKIKLAVRGAQAKRKSVYEIRNK   293
mouse           ECYVSRPTEKTVFLVFMFAVSGICVVLNLAELNHLGWRKIKLAVRGAQAKRKSVYEIRNK   300
rat             ECYVSRPTEKTVFLVFMFAVSGICVVLNLAELNHLGWRKIKLAVRGAQAKRKSVYEIRNK   300
human           ECYVSRPTEKTVFLVFMFAVSGICVVLNLAELNHLGWRKIKLAVRGAQAKRKSIYEIRNK   300
macaque         ECYVSRPTEKTVFLVFMFAVSGICVVLNLAELNHLGWRKIKLAVRGAQAKRKSIYEIRNK   300
marmoset        ECYVSRPTEKTVFLVFMFAVSGICVVLNLAELNHLGWRKIKLAVRGAQAKRKSIYEIRNK   300
cat             ECYVSRPTEKTVFLVFMFAVSGICVVLNLAELNHLGWRKIKLAVRGAQAKRKSVYEIRNK   300
pig             ECYVSRPTEKTVFLVFMFAVSGICVVLNLAELNHLGWRKIKLAVRGAQAKRKSVYEIRNK   300
guinea          ECYVSRPTEKTVFLVFMFAVSGICVVLNLAELNHLGWRKIKLAVRGAQAKRKSVYEIRNK   300
naked           ECYVSRPTEKTVFLVFMFAVSGICVVLNLAELNHLGWRKIKLAVRGAQAKRKSVYEIRNK   300
gjd2b           ECYVSRPTEKTVFLVFMFAVSGICVVLNLAELNHLGWRKIKTAVRGVQARRKSIYEIRNK   283
xenopus         ECYVSRPTEKTVFLVFMFAVSGLCVVLNLAELNHLGWRKIKMAVRGVQAKRKSIYEIRNK   283
chicken         ECYVSRPTEKTVFLVFMFAVSGICVVLNLAELNHLGWRKIKMAVRGVQAKRKSIYEIRNK   283
                **********************:****************** *:**.**:***: ***:*
```

```
gjd1a          DVSHLSSVPNLGRTQSSESAYV       299
rabbit         DLPRV-SVPNFGRTQSSDSAYV       321
dog            DLPRV-SVPNFGRT--------       306
mouse          DLPRV-SVPNFGRTQSSDSAYV       321
rat            DLPRV-SVPNFGRTQSSDSAYV       321
human          DLPRV-SVPNFGRTQSSDSAYV       321
macaque        DLPRV-SVPNFGRTQSSDSAYV       321
marmoset       DLPRV-SVPNFGRTQSSDSAYV       321
cat            DLPRV-SVPNFGRTQSSDSAYV       321
pig            DLPRV-SVPNFGRTQSSDSAYV       321
guinea         DLPRV-SVPNFGRTQSSDSAYV       321
naked          DLPRV-SVPNFGRTQSSDSAYV       321
gjd2b          DLPRM-SMPNFGRTQSSDSAYV       304
xenopus        DLPRM-GVPNFGRTQSSDSAYV       304
chicken        DLPRM-SMPNFGRTQSSDSAYV       304
               *: :: .:**:***
```

Protein sequences were retrieved from the NCBI Protein Database. Human (NP_065711.1), Mouse NP_034420.2; Rat NP_062154.1, Macaque XP_001087723.1, Marmoset JAB32260.1 Xenopus XP_002932880, Chicken NP_989913, Dog XP_038297692.1, Pig XP_020953959.1, Guinea pig XP_003475711, Naked mole (rat) XP_004861563.1, gjd2b/Cx35.1/Cx35b NP_919401.1, gjd1a/Cx34.1 NP_001122238.

Supplementary Table S1: *Cx36 mRNA expression during early development of the mouse brain*

**Structure**	**Stage**	**Assay Type**	**Reference**
brain	E14, E16, E18	NB	[1]
future brain	E7.5	RT-PCR	[2]
optic cup	E8.5	ISH	[2]
optic stalk	E8.5	ISH	[2]
forebrain	E10.5	ISH	[2]
midbrain	E10.5	ISH	[2]
thalamus	E12.5	ISH	[2]
telencephalon	E12.5	ISH	[2]
midbrain-hindbrain junction	E12.5	ISH	[2]
spinal cord	E12.5	ISH	[2]
spinal ganglion	E12.5	ISH	[2]
midbrain roof plate	E13.5	ISH	[2]
diencephalon	E13.5	ISH	[2]
main olfactory bulb	E13.5	ISH	[2]
cerebellum	E13.5	ISH	[2]
diencephalon lateral wall mantle layer	E14.5	ISH	[3]
olfactory cortex marginal layer	E14.5	ISH	[3]
telencephalon mantle layer	E14.5	ISH	[3]
metencephalon rest of alar plate mantle layer	E14.5	ISH	[3]
pons mantle layer	E14.5	ISH	[3]
ventral grey horn	E14.5	ISH	[3]
intermediate grey horn	E14.5	ISH	[3]
cervical ganglion	E14.5	ISH	[3]
cervico-thoracic ganglion	E14.5	ISH	[3]
thoracic ganglion	E14.5	ISH	[3]
spinal cord	E17.5	ISH	[2]

References:

1. Sohl, G., et al., *The murine gap junction gene connexin36 is highly expressed in mouse retina and regulated during brain development.* FEBS Lett, 1998. **428**(1-2): p. 27-31.
2. Gulisano, M., et al., *Cx36 is dynamically expressed during early development of mouse brain and nervous system.* Neuroreport, 2000. **11**(17): p. 3823-8.
3. Diez-Roux, G., et al., *A high-resolution anatomical atlas of the transcriptome in the mouse embryo.* PLoS Biol, 2011. **9**(1): p. e1000582.

Supplementary Table S2: *Cx36 mRNA and protein expression in the adult rodent nervous system*

Tissue	Subdivsion	RNA	Protein	reference
Telencephalon	cortex	high density labelling in layers IV, V and VI	pyramidal neurons, GABAergic interneurons<br>PV positive interneurons	[1]<br>[2] [3-5]
	olfactory system including olfactory epithelium, accessory systems	mitral cell layer, glomerular layer, external plexiform layer, periglomerular cells, tufted cells, granular cell layer, olfactory epithelial layer	olfactory nerve bundles underlying the olfactory epithelium, olfactory nerve layer, glomerular layer of the olfactory bulb, accessory olfactory bulb, vomeronasal nerve, gap junctions between mitral cells	[1] [2, 6-9]
	hippocampus	all regions of the hippocampal formation, including the enthorinal cortex	CA3/4, mossy fiber terminals	[1, 2, 6, 7, 10-12]
	amygdala	medial amygdaloid nucleus, the anterior and posteromedial cortical amygdaloid nucleus, the amygdalo-hippocampal transition area, medial and lateral part of the central nucleus	basomedial nuclei<br>basolateral nuclei	[7, 13]
	basal ganglia	caudate putamen,<br>globus pallidus and nucleus accumbens labeling was localized<br>in a subpopulation of scattered cells with large pale nuclei.		[1, 14]
Diencephalon	thalamus	all major nuclei, reticular thalamic nucleus, including subthalamic nucleus	reticular thalamic nucleus	[13, 15]
	hypothalamus	lateral and anterior arcuate nucleus		[15] [7]

	other midbrain structures	nuclei of the preoptic region, of the anterior region of the paraventricular nucleus, anterior hypothalamic nucleus, lateral hypothala-mic area, supraoptic nucleus, of the tuberal region such as arcuate nucleus, ventromedial and dorsomedial nucleus, perifornical nucleus. Mammillary region.		[7, 15]
	habenular nuclei, pineal gland	medial habenular nucleus, scattered in the lateral nucleus. intense signal in the pineal gland	tryptophan hydroxylase-positive and 5-hydroxytryptamine-positive pinealocytes	[1, 6, 16]
	pituitary gland	no data	anterior pituitary gland	[1, 17]
	retina	ganglion cell layer and in the inner border of the inner nuclear layer	AII amacrine cells; ganglion cells, bipolar cells, photoreceptor	[1] [2, 6, 18-24] [25-28]
Hindbrain	cerebellum	molecular layer, granular layer, deep nuclei	molecular layer<br>Purkinje neurons	[29]<br>[1, 2, 6]
	inferior olive	all olivary neurons	all olivary neurons	[1] [2, 6, 18, 22]
	pons	raphe, trochlear, reticulotecemental motor trigeminal basilar pontine	trigeminal ganglion	[30]
	medulla	most nuclei such as the red, pararubral and oculomotor nuclei, the periaqueductal grey and the superior and inferior colliculi	lacZ transgenic	[31]

Spinal cord	spinal cord	all the lamina of the gray matter of the spinal cord, with the highest grain density in the motor neurons of the anterior horn	detected in the IML, IC and CA and in laminae VIII and IX<br>ventral horn	[18]
	ganglions	dorsal root ganglia	dorsal root ganglia<br>lumbar dorsal root; trigeminal sensory ganglia	[32, 33]
Other	neural crest-derived neuroendocrine organ	carotid body	carotid body	[34]
	neural crest-derived neuroendocrine organ	myenteric plexus	myenteric plexus	[34]

References

1. Belluardo, N., et al., *Expression of connexin36 in the adult and developing rat brain.* Brain Res, 2000. **865**(1): p. 121-38.
2. Teubner, B., et al., *Functional expression of the murine connexin 36 gene coding for a neuron-specific gap junctional protein.* J Membr Biol, 2000. **176**(3): p. 249-62.
3. Meier, C., et al., *Immunohistochemical detection of the neuronal connexin36 in the mouse central nervous system in comparison to connexin36-deficient tissues.* Histochem Cell Biol, 2002. **117**(6): p. 461-71.
4. Ma, Y., et al., *Expression of gap junction protein connexin36 in multiple subtypes of GABAergic neurons in adult rat somatosensory cortex.* Cereb Cortex, 2011. **21**(11): p. 2639-49.
5. Priest, C.A., A.J. Thompson, and A. Keller, *Gap junction proteins in inhibitory neurons of the adult barrel neocortex.* Somatosens Mot Res, 2001. **18**(4): p. 245-52.
6. Condorelli, D.F., et al., *Cloning of a new gap junction gene (Cx36) highly expressed in mammalian brain neurons.* Eur J Neurosci, 1998. **10**(3): p. 1202-8.
7. Parenti, R., et al., *Expression of connexin36 mRNA in adult rodent brain.* Neuroreport, 2000. **11**(7): p. 1497-502.
8. Zhang, C. and D. Restrepo, *Heterogeneous expression of connexin 36 in the olfactory epithelium and glomerular layer of the olfactory bulb.* J Comp Neurol, 2003. **459**(4): p. 426-39.
9. Christie, J.M., et al., *Connexin36 mediates spike synchrony in olfactory bulb glomeruli.* Neuron, 2005. **46**(5): p. 761-72.

10. Nagy, J.I., *Evidence for connexin36 localization at hippocampal mossy fiber terminals suggesting mixed chemical/electrical transmission by granule cells.* Brain Res, 2012. **1487**: p. 107-22.
11. Hamzei-Sichani, F., et al., *Mixed Electrical-Chemical Synapses in Adult Rat Hippocampus are Primarily Glutamatergic and Coupled by Connexin-36.* Front Neuroanat, 2012. **6**: p. 13.
12. Condorelli, D.F., et al., *Cellular expression of connexins in the rat brain: neuronal localization, effects of kainate-induced seizures and expression in apoptotic neuronal cells.* Eur J Neurosci, 2003. **18**(7): p. 1807-27.
13. Rash, J.E., et al., *High-resolution proteomic mapping in the vertebrate central nervous system: close proximity of connexin35 to NMDA glutamate receptor clusters and co-localization of connexin36 with immunoreactivity for zonula occludens protein-1 (ZO-1).* J Neurocytol, 2004. **33**(1): p. 131-51.
14. Venance, L., J. Glowinski, and C. Giaume, *Electrical and chemical transmission between striatal GABAergic output neurones in rat brain slices.* J Physiol, 2004. **559**(Pt 1): p. 215-30.
15. Lassen, M.B., et al., *Brain stimulation reward is integrated by a network of electrically coupled GABA neurons.* Brain Res, 2007. **1156**: p. 46-58.
16. Wang, S.G., et al., *Connexin36 localization to pinealocytes in the pineal gland of mouse and rat.* Eur J Neurosci, 2017. **45**(12): p. 1594-1605.
17. Gongrich, C., et al., *Electrotonic Coupling in the Pituitary Supports the Hypothalamic-Pituitary-Gonadal Axis in a Sex Specific Manner.* Front Mol Neurosci, 2016. **9**: p. 65.
18. Rash, J.E., et al., *Immunogold evidence that neuronal gap junctions in adult rat brain and spinal cord contain connexin-36 but not connexin-32 or connexin-43.* Proc Natl Acad Sci U S A, 2000. **97**(13): p. 7573-8.
19. Al-Ubaidi, M.R., et al., *Functional properties, developmental regulation, and chromosomal localization of murine connexin36, a gap-junctional protein expressed preferentially in retina and brain.* J Neurosci Res, 2000. **59**(6): p. 813-26.
20. Guldenagel, M., et al., *Visual transmission deficits in mice with targeted disruption of the gap junction gene connexin36.* J Neurosci, 2001. **21**(16): p. 6036-44.
21. Hidaka, S., T. Kato, and E. Miyachi, *Expression of gap junction connexin36 in adult rat retinal ganglion cells.* J Integr Neurosci, 2002. **1**(1): p. 3-22.
22. Meier, C., et al., *Immunohistochemical detection of the neuronal connexin36 in the mouse central nervous system in comparison to connexin36-deficient tissues.* Histochemistry and Cell Biology, 2002. **117**(6): p. 461-471.
23. Feigenspan, A., et al., *Expression of connexin36 in cone pedicles and OFF-cone bipolar cells of the mouse retina.* J Neurosci, 2004. **24**(13): p. 3325-34.
24. Dedek, K., et al., *Localization of heterotypic gap junctions composed of connexin45 and connexin36 in the rod pathway of the mouse retina.* Eur J Neurosci, 2006. **24**(6): p. 1675-86.

25. Han, Y. and S.C. Massey, *Electrical synapses in retinal ON cone bipolar cells: subtype-specific expression of connexins.* Proc Natl Acad Sci U S A, 2005. **102**(37): p. 13313-8.
26. Volgyi, B., et al., *Morphology and tracer coupling pattern of alpha ganglion cells in the mouse retina.* J Comp Neurol, 2005. **492**(1): p. 66-77.
27. Li, H., et al., *Adenosine and dopamine receptors coregulate photoreceptor coupling via gap junction phosphorylation in mouse retina.* J Neurosci, 2013. **33**(7): p. 3135-50.
28. Kantor, O., et al., *Characterization of connexin36 gap junctions in the human outer retina.* Brain Struct Funct, 2016. **221**(6): p. 2963-84.
29. Nagy, J.I. and J.E. Rash, *Cx36, Cx43 and Cx45 in mouse and rat cerebellar cortex: species-specific expression, compensation in Cx36 null mice and co-localization in neurons vs. glia.* Eur J Neurosci, 2017. **46**(2): p. 1790-1804.
30. Garrett, F.G. and P.L. Durham, *Differential expression of connexins in trigeminal ganglion neurons and satellite glial cells in response to chronic or acute joint inflammation.* Neuron Glia Biol, 2008. **4**(4): p. 295-306.
31. Degen, J., et al., *Expression pattern of lacZ reporter gene representing connexin36 in transgenic mice.* J Comp Neurol, 2004. **473**(4): p. 511-25.
32. Perez Armendariz, E.M., et al., *Neurons and satellite glial cells in adult rat lumbar dorsal root ganglia express connexin 36.* Acta Histochem, 2018. **120**(3): p. 168-178.
33. Nagy, J.I., et al., *Connexin36 Expression in Primary Afferent Neurons in Relation to the Axon Reflex and Modality Coding of Somatic Sensation.* Neuroscience, 2018. **383**: p. 216-234.
34. Frinchi, M., et al., *Connexin36 (Cx36) expression and protein detection in the mouse carotid body and myenteric plexus.* Acta Histochem, 2013. **115**(3): p. 252-6.
